# Analysis of Tool Wear in Finish Turning of Titanium Alloy Ti-6Al-4V Under Minimum Quantity Lubrication Conditions Observed with Recurrence Quantification Analysis

**DOI:** 10.3390/ma18010079

**Published:** 2024-12-27

**Authors:** Joanna Lisowicz, Krzysztof Krupa, Kamil Leksycki, Rafał Rusinek, Szymon Wojciechowski

**Affiliations:** 1 Department of Manufacturing Techniques and Automation, Faculty of Mechanical Engineering and Aeronautics, Rzeszow University of Technology, 12 Al. Powstancow Warszawy Street, 35-959 Rzeszow, Poland; 2 Department of Material Science, Faculty of Mechanical Engineering and Aeronautics, Rzeszow University of Technology, 12 Al. Powstancow Warszawy Street, 35-959 Rzeszow, Poland; krupa@prz.edu.pl; 3 Institute of Mechanical Engineering, University of Zielona Gora, 4 Prof. Z. Szafrana Street, 65-516 Zielona Gora, Poland; 4 Department of Applied Mechanics, Lublin University of Technology, Nadbystrzycka 36 Street, 20-618 Lublin, Poland; r.rusinek@pollub.pl; 5 Institute of Mechanical Technology, Poznan University of Technology, 3 Piotrowo Street, 60-965 Poznan, Poland; szymon.wojciechowski@put.poznan.pl; 6 Department of Automobile and Manufacturing Technologies, Faculty of Manufacturing Technologies with a Seat in Presov, Technical University of Kosice, 31 Sturova Street, 080 01 Presov, Slovakia

**Keywords:** recurrence plots, recurrence quantification analysis, titanium alloy, minimum quantity lubrication, turning

## Abstract

Titanium alloys, particularly Ti-6Al-4V, are widely used in many industries due to their high strength, low density, and corrosion resistance. However, machining these materials is challenging due to high strength at elevated temperatures, low thermal conductivity, and high chemical reactivity. This study investigates Recurrence Plot (RP) and Recurrence Quantification Analysis (RQA) to analyze tool wear during the finish turning of Ti-6Al-4V. The tests were conducted under Minimum Quantity Lubrication (MQL). Three inserts (two coated, one uncoated) were tested, and tool life was evaluated based on material removal volume. The issue of tool exploitation and process reliability is crucial, as it directly impacts machining performance. Results show that the uncoated insert outperformed the coated ones. RQA parameters indicated a stable-to-unstable transition in coated inserts but not in the uncoated insert. This suggests that recurrence analysis can monitor cutting dynamics in coated insert machining, but further research is needed for uncoated tools. This paper’s novelty lies in applying RP and RQA to diagnose tool wear in titanium alloy machining under MQL conditions, a method not previously explored in this context.

## 1. Introduction

Titanium alloys find broad application in industries such as automotive, aerospace, and medicine, owing to their unique combination of properties, including high strength, low density, resistance to corrosion, and stability at elevated temperatures [1]. Despite these advantages, titanium alloys present significant challenges in machining [2]. One of the primary difficulties is their low thermal conductivity, which hampers effective heat dissipation from the cutting zone, resulting in rapid tool wear and surface degradation of machined parts. Additionally, titanium’s high chemical reactivity at elevated temperatures can cause adverse interactions with cutting tools, leading to adhesion, galling, and premature wear. Furthermore, titanium alloys retain considerable strength even at elevated temperatures, making them more difficult to machine compared to metals like aluminum or steel. Another challenge is their tendency to undergo work hardening during machining, where the material becomes harder with each pass, exacerbating tool wear and complicating dimensional accuracy [3,4].

Considering these challenges, the machining of titanium alloys requires careful consideration of tool materials [5,6], tool geometry [7,8], cutting parameters [9], and cooling strategies [10]. Cooling is crucial when machining titanium alloys. Dry machining leads to higher cutting temperatures, workpiece hardening, and faster tool wear. The most commonly used cooling strategy in the industry is flood cooling. Nevertheless, maximum machining efficiency can be attained through the use of high-pressure cooling (HPC) [11,12].

On the other hand, it is well known that most coolants used in machining are toxic, non-biodegradable, and expensive to dispose of. Therefore, intensive research is being conducted on the application of Minimum Quantity Lubrication (MQL) and Minimum Quantity Cooling and Lubrication (MQCL) in the machining of titanium alloys. In these methods, a small amount of lubricant or coolant, respectively (10–500 mL/h), is dispersed in an airflow aimed at the cutting zone, significantly reducing the amount of coolant and lubricant used compared to flood and high-pressure cooling [13,14].

Scientific studies are investigating the influence of various liquids [15], the flow rate of these liquids [16,17], and the addition of nanoparticles [18,19] on tool wear, cutting forces, and the surface roughness of the machined parts.

An important issue in assessing the machining process is the choice of methods for analyzing phenomena that occur during machining. Examples of methods based on signal analysis include recurrence plots (RPs) and Recurrence Quantification Analysis (RQA) [20]. The Recurrence Plot (RP) method offers a qualitative analysis of underlying patterns in dynamical systems by reconstructing the system’s phase space through time-delay embedding of the data. Initially introduced by Eckmann et al. [21], an RP is visualized as a square matrix where points indicate moments when the system revisits a previous state. Technically, an RP highlights each moment (represented by time axes *i* and *j*) when the phase space trajectory returns to approximately the same region. The parameters *m*, *d*, and *ε* represent the embedding dimension, time delay, and threshold, respectively, with detailed guidance on selecting these parameters provided in [22,23].

The RP method has evolved to include quantitative measures derived from points and lines within the plot, known as Recurrence Quantification Analysis (RQA). RQA is a nonlinear analysis method that quantifies recurrence patterns in a system’s state space. Zbilut and Webber [24] introduced various complexity measures for analyzing small-scale structures in RPs, based on the density of recurrence points and the patterns formed by diagonal and vertical lines. More comprehensive information on the above-mentioned methods can be found in [22].

**Figure 1:**
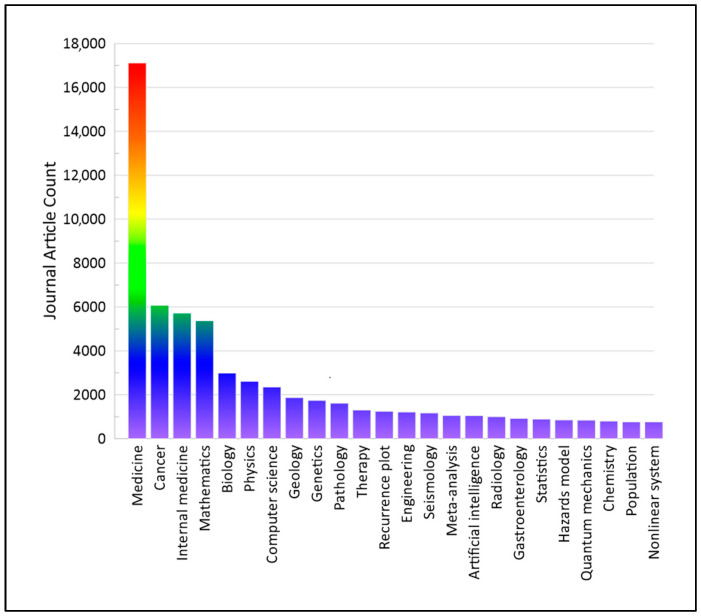
Number of journal articles containing the keyword “recurrence plot”, divided into scientific fields, published from 1980 to 2024 (based on data from Lens.org).

The possibilities of using the above-described methods are widely studied in medicine and many other fields of science, as shown graphically in Figure 1, based on data from Lens.org. Figure 1 presents the number of journal articles containing the keywords “recurrence plot” across different scientific fields, published from 1980 to 2024.

In medicine, recurrence plots are used to analyze signals from the human body to assess health status or the effectiveness of therapy. Examples of the use of RP and RQA in medicine include distinguishing persistent and paroxysmal atrial fibrillation based on complex fractionated atrial electrograms (CFAEs) [25]; analyzing sleep stages using EEG signals [26]; assessing seizure states in epileptic patients based on intracerebral recordings [27]; diagnosing and grading Parkinson’s disease severity owing to vertical ground reaction force signals during clinical gait measurements [28]; and analyzing long-term measurements of cerebral tissue oxygen saturation (StO_2_), arterial oxygen saturation (SpO_2_), fractional tissue extraction (FTOE), and heart rate (HR) in premature infants [29]. Additionally, RP and RQA have been used to evaluate the effects of functional electrical stimulation (FES) therapy in hemiplegic patients [30].

**Figure 2:**
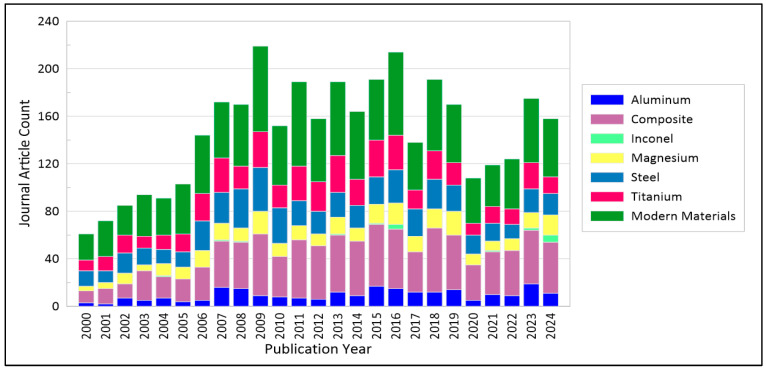
Number of journal articles using recurrence techniques for the analysis of the cutting process, divided by machined material, published from 2000 to 2024 (based on data from Lens.org).

According to the existing research findings, it can be inferred that the recurrence plot and RQA techniques are employed to analyze the turning and milling processes of various materials: composite materials [31,32], glass [33], superalloys (e.g., titanium alloys, Inconel), magnesium alloys, and other types of metal materials that are presented graphically in Figure 2. Analyzing the popularity of recurrence techniques since 2000, one can observe an increasing trend in the cutting of composites and other modern materials.

Chen et al. [34] analyzed the milling of aluminum alloy 7075-T651 and found that machining state changes can be reflected by three features—Entropy (*ENT*), Maximum Diagonal Length (*L_max_*), and Recurrence Time of First Typing (*RTFT*)—which provide a basis for monitoring vibrations under varying conditions. Radhakrishnan et al. [35] showed that RQA can estimate flank wear based on force measurements when turning AISI 6150 steel with uncoated carbide tools. Gangadhar et al. [36] applied RQA successfully to diagnose faults in WC inserts while turning die steel. Kamarthi et al. [37] concluded that recurrence statistics of acoustic emission signals during the turning of AISI 1054 steel contain characteristic information about surface roughness. Govindan and Namboothiri [38] demonstrated that RQA can effectively identify the shift from chatter-free to chatter-induced cutting in turning, with the RQA parameter "percent determinism" rising as chatter occurs.

Rusinek et al. [39] analyzed experimental data from milling the titanium super-alloy Ti6242 using RP and the Hilbert–Huang transform. They concluded that classical modal analysis is ineffective for predicting instabilities in the milling of titanium alloys. Certain RQA parameters, such as Determinism-to-Recurrence Ratio (*DET/RR*), Entropy (*ENT*), Laminarity-to-Determinism Ratio (*LAM/DET*), Ventricle (*V_ent_*), second-order recurrence time (*T_2_)*, and Maximum Vertical Length (*V_max_*), were found useful in identifying stable and unstable states of the cutting process.

Rusinek [40] compared the cutting process of titanium alloy Ti-6Al-4V, stainless steel EZ6NCT25, and constructional steel C45. Cutting forces and workpiece displacements were measured as a function of the depth of cut. Based on RPs, it was concluded that machining with small cutting depths is characterized by regular vibrations. The highest vibration levels, measured as the standard deviation of displacement, were observed at a 0.8 mm depth of cut for the titanium alloy, likely due to friction phenomena and the material’s lower Young’s modulus compared to steel. Additionally, it was observed that better results could be achieved by analyzing displacement time series rather than cutting force data, due to the influence of stochastic components.

In summary, titanium alloys are broadly used in many sectors, but their properties make them difficult to machine. Given the broad applications of these alloys, RP and RQA methods should be considered for analyzing machining processes. The issue of tool exploitation and process reliability plays an essential role in ensuring the efficiency and consistency of machining operations, especially when working with challenging materials like titanium alloys. A review of the literature revealed no studies applying these methods to Ti-6Al-4V turning under MQL conditions. Therefore, this study investigates the finish turning of Ti-6Al-4V under MQL conditions using three different cutting inserts. During machining, three components of the total cutting force were measured, tool life was evaluated, and recurrence plots were generated. Based on these plots, the RQA parameters—Recurrence Rate (*RR*), Determinism (*DET*), Average Diagonal Line Length (*L*), Maximum Diagonal Length (*L_max_*), Entropy (*ENT*), Trapping Time (*TT*), and Maximum Vertical Length (*V_max_*)—were analyzed.

Taking into account all the above, the main aim of this study is to estimate the possibility of using recursive techniques to diagnose tool wear when cutting titanium alloy under MQL conditions.

## 2. Materials and Methods

**Figure 3:**
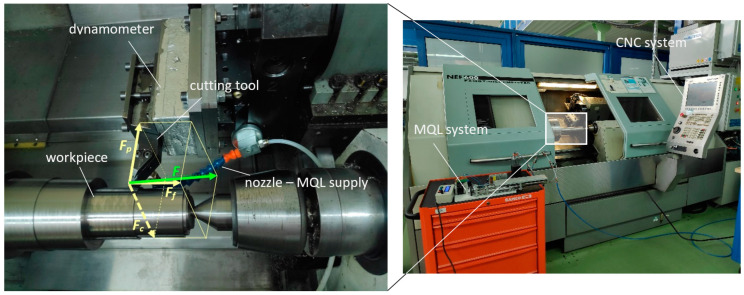
Workstation—NEF 600 lathe with MQL system.

Turning tests were performed on a NEF 600 lathe (Gildemeister AG, Bielefeld, Germany)—Figure 3. The machined material was a two-phase titanium alloy Ti-6Al-4V. The test samples were provided as rods with a diameter of 100 mm.

Three positive rhombic cutting inserts were selected for the tests:

- VCMT160404 IC907 (ISCAR Ltd., Tefen, Israel; referred to hereafter as cutting insert A): a cutting insert made from a tough sub-micron substrate, PVD-coated with TiAlN + TiN;
- VBGT160404 1115 (Sandvik Coromant, Sandviken, Sweden; referred to hereafter as cutting insert B): a fine-grained carbide insert, PVD-coated with TiAlN + TiAlN;
- VBGT160404-M3 HX (Seco Tools, Fagersta, Sweden; referred to hereafter as cutting insert C): an uncoated insert made from HX sintered carbide.

SVJBL2525M16 JET (Seco Tools, Fagersta, Sweden) tool holder with the possibility of an internal coolant/oil mist supply was used to mount the inserts. Turning was performed under Minimum Quantity Lubrication (MQL) conditions using a liquid blend of vegetable oil and diester. Liquid flow rate was 30 mL/h and air pressure was 0.7 MPa. Table 1 provides a summary of the experimental setup and cutting parameters.

During the experiment, three components of cutting force were measured by means of a Kistler 9257B piezoelectric dynamometer (Kistler Instrumente AG, Winterthur, Switzerland).

In this study, all RQA measures were evaluated and seven were selected for their effectiveness in assessing stability in turning processes. These include Recurrence Rate (*RR*), which calculates the ratio of recurrence points to all possible points in the Recurrence Plot (RP), and Determinism (*DET*), which is the proportion of recurrence points forming diagonal lines. Other key measures include averaged diagonal line length (*L*), Maximum Diagonal Length (*L_max_*), Entropy (*ENT*), Trapping Time (*TT*), and the longest vertical line (*V_max_*).

To conduct RQA, the average mutual information function and the false nearest neighbors method were used to estimate the embedding parameters *m* and *d,* respectively, as described in [23]. These parameters were used to generate recurrence plots (RPs), which depict instances when the trajectory in phase space returns to a nearly identical region. Mathematically, an RP is represented by a matrix:

(1) $$M_{ij}=\theta \left(\varepsilon -\left|s_{i}-s_{j}\right|\right),$$

where *θ* is the Heaviside step function, *ε* is a threshold parameter, and *s_i_* and *s_j_* are delay vectors. If recurrence appears then *M_ij_* = 1, which in the RP visualization is plotted as black dots, otherwise *M_ij_* = 0—white dots in the RP visualization.

An alternative method, used in this paper, is the unthreshold recurrence plot (UTRP) that represents the distances between each state vector s(i), corresponding to time i, and all other vectors in the phase space. This is expressed as Dist(i,j)=ϑ(∥s(i)−s(j)∥), where ∥⋅∥ denotes a distance measure (such as the Euclidean norm), and ϑ(⋅) is a color mapping function that assigns a specific color to each distance. However, analyzing visual patterns in RPs or UTRPs may be subjective; therefore, RQA is often used to statistically describe these plots [24]. The definitions of the RQA indicators used in this study are as follows:

- Recurrence Rate (*RR*) that represents the proportion of recurrent points in the RPs to the total number of possible point pairs:

(2) $$RR=\frac{1}{N^{2}}\sum_{i,j,=1}^{N}R_{i,j}$$

- Determinism (*DET*) measures the percentage of recurrent points forming diagonal lines in the plot:

(3) $$DET=\frac{\sum_{l=lmin}^{N}lP\left(l\right)}{\sum_{l=1}^{N}lP\left(l\right)}$$

- Average diagonal line length (*L*), which is the mean length of all diagonal lines in the recurrence plot:

(4) $$L=\frac{\sum_{l=l_{min}}^{N}lP\left(l\right)}{\sum_{l=l_{min}}^{N}P\left(l\right)}$$

- Maximum diagonal line length (*L_max_*) is the longest diagonal line in the recurrence plot:

(5) $$L_{max}=max\left(\left\{l_{i};i=1,\;\ldots ,N_{l}\right\}\right)$$

- Entropy (*ENT*) represents the Shannon entropy of the distribution of diagonal line lengths in the recurrence plot:

(6) $$ENT=-\sum_{l=lmin}^{N}P\left(l\right)\;lnP\left(l\right)$$

- Trapping time (*TT*) is the average length of the vertical lines in the RPs:

(7) $$TT=\frac{\sum_{v=v_{min}}^{N}vP\left(v\right)}{\sum_{v=v_{min}}^{N}P\left(v\right)}$$

- Length of the longest vertical line (*V_max_*):

(8) $$V_{max}=\max\left(\left\{v_{i};i=1,\ldots ,N_{v}\right\}\right)$$

where *P*(*l*) is the histogram of diagonal line lengths *l*. *P*(*v*) is the histogram of vertical line lengths *v*, and *N* denotes the number of points in the phase space trajectory.

In this study, an application developed by H. Yang [41] was used to generate recurrence plots based on cutting force component data, and RQA parameters were calculated with the use of an application created by J. Zubek [42].

## 3. Results and Discussion

The machining was performed using 50 mm tool passes. Since turning was carried out on different diameters of the workpiece, tool life was measured based on the volume of removed material until the tool wear criterion was achieved, allowing for comparison between the three cutting inserts.

### 3.1. Cutting Force Components and Tool Life

**Figure 4:**
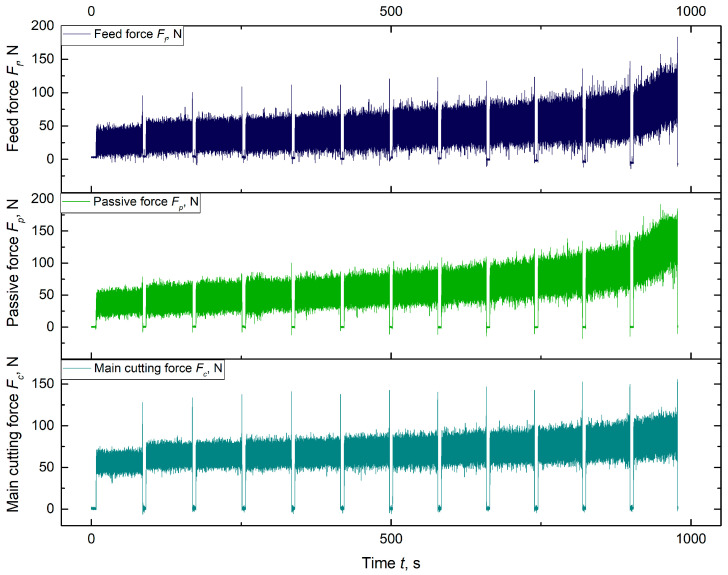
Record of cutting force components during turning on the example of cutting insert C.

The tool wear criterion was defined as a tool wear indicator *VB_C_* ≈ 0.2 mm for uncoated tools and 0.3 mm for coated tools. Above these limit values, a rapid increase in the values of the cutting force components was observed, and the surface quality of the machined part deteriorated rapidly. The *VB_C_* index represents the width of the wear band in zone C, which includes the rounded part of the corner. This index is particularly effective in describing blade wear when the tool corner radius exceeds the cutting depth [43,44]. According to previous research [45], it was observed that the above-mentioned values of *VB_C_* indicator correspond to a value of passive force component *F_p_* of 125 N. Kistler 9257B piezoelectric dynamometer was used to measure cutting force components (the example of recorded cutting force components is shown in Figure 4). Since the experiment was conducted as the longitudinal turning of 50 mm segments of a shaft, the recorded signal of the individual cutting force components reveals stages where the tool was in contact with the material, as well as stages where the tool retracted and re-engaged with the material (values oscillating around zero). For the example presented in Figure 4, 12 passes of 50 mm were recorded for tool C. However, since the machining was performed on different diameters of the workpiece, the analysis of tool durability was based on the volume of material removed, as previously mentioned.

**Figure 5:**
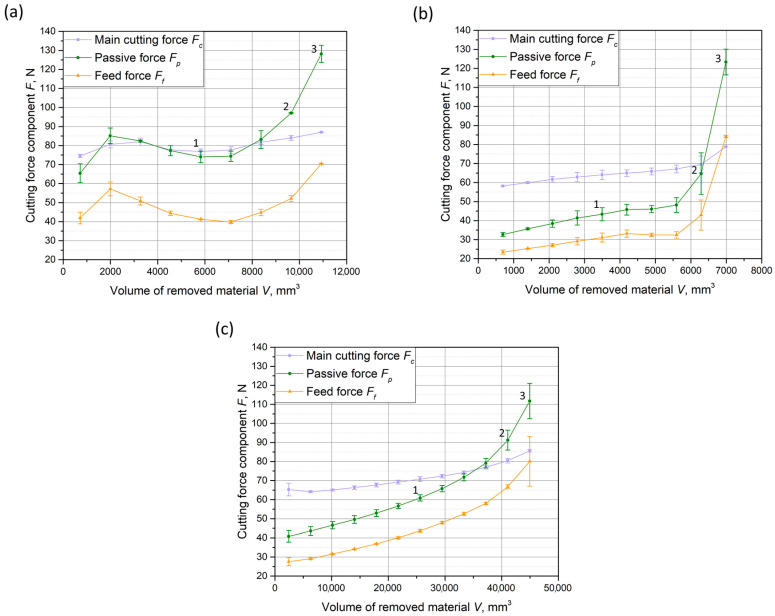
Mean value of cutting force components in the function of the volume of removed material for (**a**) cutting insert A, (**b**) cutting insert B, and (**c**) cutting insert C.

**Figure 6:**
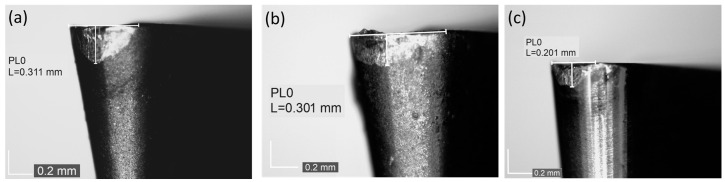
Images of (**a**) cutting insert A, (**b**) cutting insert B, and (**c**) cutting insert C after achieving the tool wear criterion (point 3 in Figure 5).

Figure 5 illustrates the changes in the mean values of the components of the cutting force as the volume of material removed increases during turning. Initially, the highest value was observed for the main cutting force component, *F_c_*, while the feed force component, *F_f_*, was the lowest. As the machining process progressed, tool wear occurred (resulting in changes to its microgeometry), leading to an increase in the values of the individual cutting force components for all three cutting inserts. The most significant increase was observed in the passive force, *F_p_*. Points 1, 2, and 3 are marked to indicate key moments in the process. Point 1 corresponds to the middle of the cutting operation, while points 2 and 3 represent the final measurements during tool life. These points were used later in the article to present the changes in the recurrence plots and analyze the dynamics of tool wear at different stages. Figure 6 shows images of the worn cutting inserts, corresponding to the point marked as 3 in Figure 5, after the tool wear criterion was reached.

**Figure 7:**
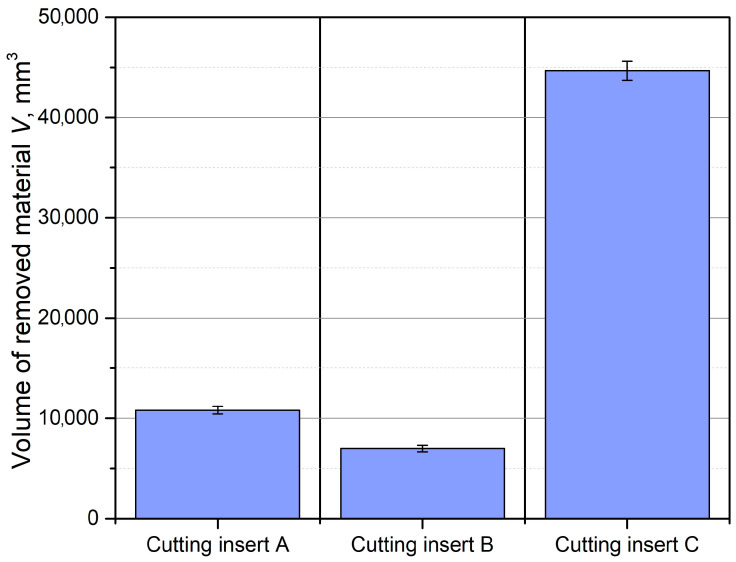
Volume of removed material for each cutting insert.

A summary of the material removal volume for each cutting insert, until the tool life criterion *VB_C_* was reached, is shown in Figure 7. It is evident that the greatest tool life was achieved with cutting insert C, which lasted more than three times longer than insert A and more than five times longer than insert B. This can be attributed to the fact that insert C was uncoated, allowing for a sharper cutting geometry and edge. In contrast, inserts A and B were coated, which introduced a rounding radius on the cutting edge. When machining difficult-to-cut materials, such as the titanium alloy used in this study, less sharp tools lead to workpiece surface hardening. As a result, subsequent layers of material become harder, causing an increase in cutting forces and a reduction in tool life.

### 3.2. Recurrence Plots and RQA Analysis

**Figure 8:**
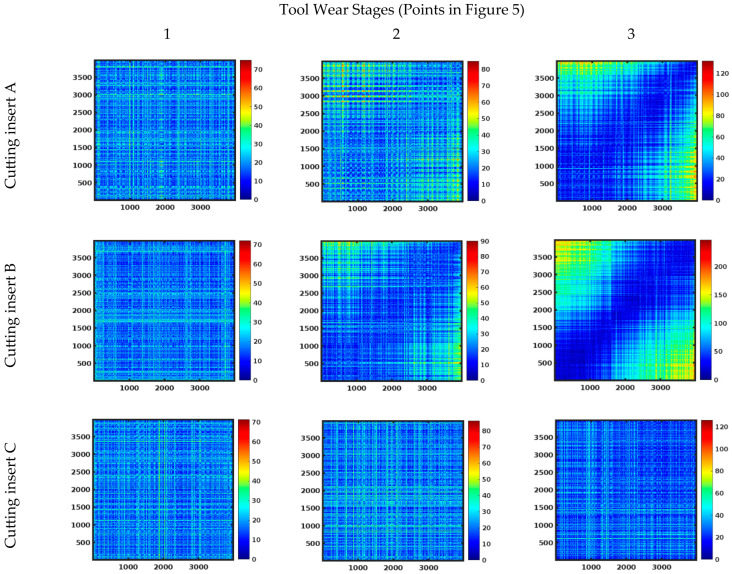
Unthreshold recurrence plots for subsequent stages of tool wear.

Figure 8 presents selected unthreshold recurrence plots corresponding to points 1, 2, and 3 in Figure 5. When the color code is blue, it indicates that the points are close to each other in the record of cutting force components, while a red color signifies that the points are farther apart [46].

For cutting inserts A and B, the recurrence plots observed throughout the machining process were similar to those obtained at point 1 (marked in Figure 5), showing homogeneous RP graphs typical of stationary systems. These graphs closely resemble those obtained in previous studies, particularly the ones for the passive force [47]. Towards the end of tool life (points 2 and 3), a gradual increase in the concentration of blue points along the main diagonal was observed for cutting inserts A and B. This indicates a transition from a stable to an unstable state. In contrast, for cutting insert C, no clear difference was observed in the form of the recurrence plot. Only the upper right corner of the graph showed a greater concentration of blue dots, indicating a higher density of points.

Based on the recurrence plots for the tested cutting inserts, it can be concluded that the method is useful for identifying the moment when the critical tool wear threshold is reached. However, for uncoated tools, the results are not entirely conclusive. Therefore, it would be advisable to complement this approach with image analysis using artificial intelligence to improve accuracy.

**Figure 9:**
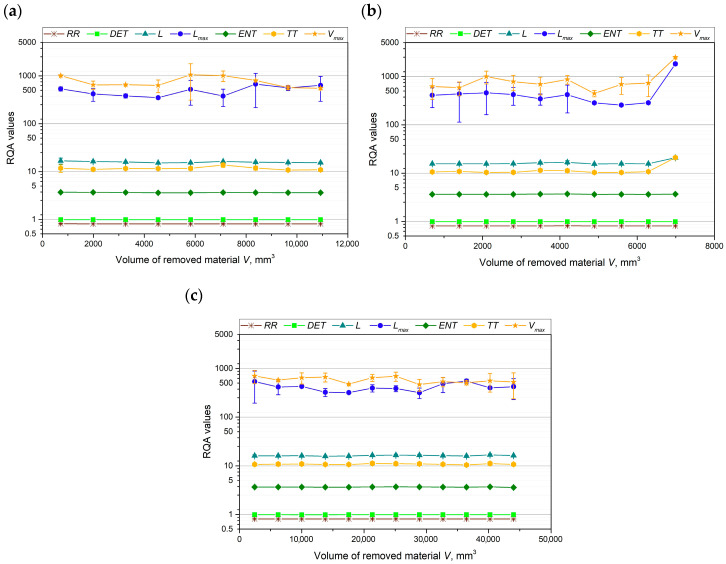
RQA parameters on the basis of analysis of the records of main cutting force component *F_c_* for (**a**) cutting insert A, (**b**) cutting insert B, and (**c**) cutting insert C.

**Figure 10:**
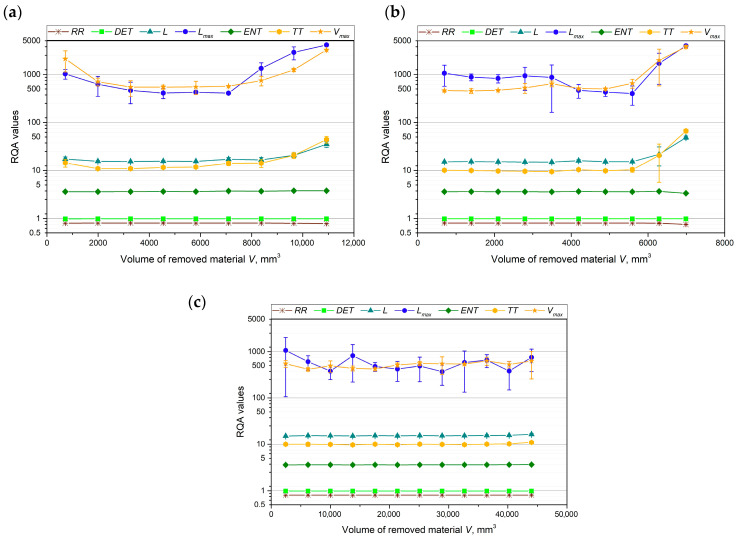
RQA parameters on the basis of analysis of the records of main cutting force component *F_p_* for (**a**) cutting insert A, (**b**) cutting insert B, and (**c**) cutting insert C.

**Figure 11:**
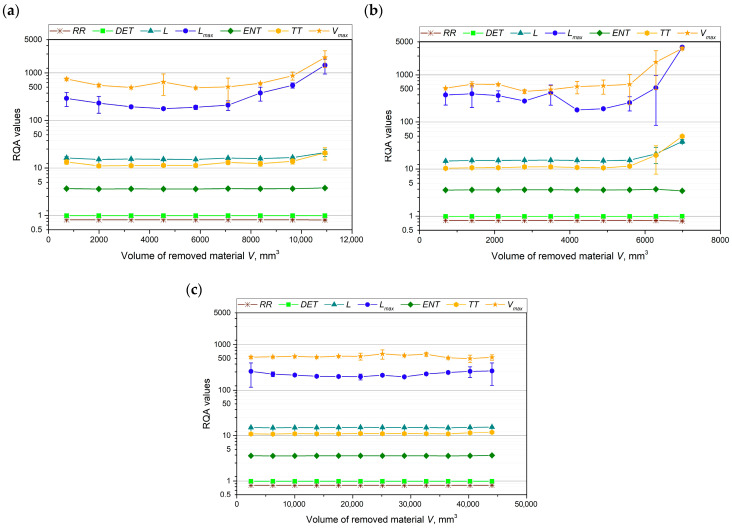
RQA parameters on the basis of analysis of the records of main cutting force component *F_f_* for (**a**) cutting insert A, (**b**) cutting insert B, and (**c**) cutting insert C.

To further analyze the curves of the tested cutting force components, an evaluation of the RQA parameters was also performed. It has been previously reported in the literature [46] that, based on the analysis of RQA parameters in the machining process, chatter-free cutting can be identified, and chatter begins once a certain level of tool wear is reached. Figure 9, Figure 10 and Figure 11 show how the values of the RQA parameters changed throughout the turning process, depending on the volume of material removed, for each cutting force component and cutting insert.

The values of the *RR*, *DET*, and *ENT* parameters remained constant throughout the tool life for all three cutting force components and all the cutting inserts tested. In the case of the main cutting force component, *F_c_* (Figure 9), no clear correlation was observed between the remaining RQA parameters and the volume of material removed for cutting inserts A and C. However, for cutting insert B, an evaluation of the *L_max_* and *V_max_* parameters allowed for the distinction of three distinct phases: the tool run-in period, the stable machining period, and the rapid tool wear period.

The most distinct relationship between the RQA parameters—*L*, *L_max_*, *TT*, and *V_max_*—and tool wear can be observed for cutting inserts A and B, particularly with the passive and feed force components (Figure 10a,b and Figure 11a,b). The values of *TT* and *L* remained constant throughout the machining process, with an increase in these parameters occurring only at the end of the tool’s life. In contrast, for cutting insert B, the *L_max_* and *V_max_* parameters clearly indicated a tool lapping period. For cutting insert C, no significant relationship between the RQA parameters and tool wear was observed.

**Figure 12:**
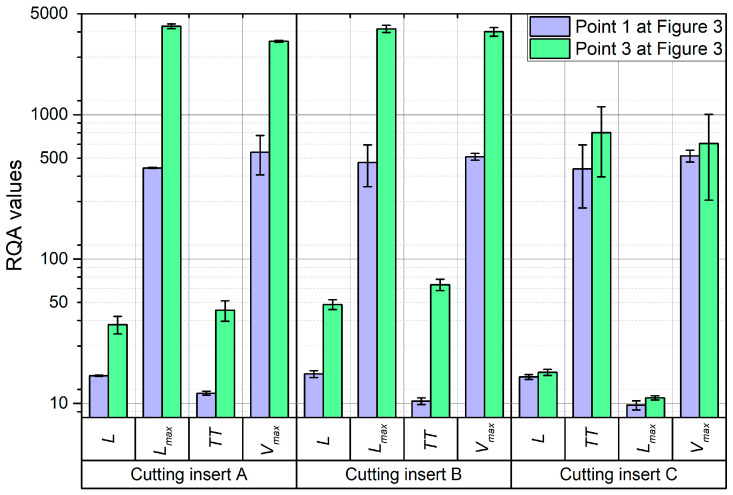
Comparison of selected RQA parameters determined on the basis of the recording of passive force for stable machining (point 1 in Figure 5) and for the end of machining (point 3 in Figure 5).

**Figure 13:**
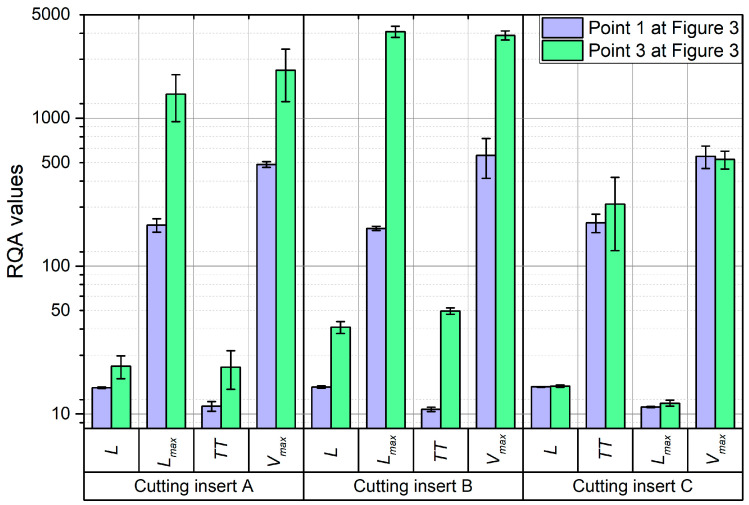
Comparison of selected RQA parameters determined on the basis of the recording of feed force for stable machining (point 1 in Figure 5) and for the end of machining (point 3 in Figure 5).

Since the values of the parameters *L*, *L_max_*, *TT*, and *V_max_* for the passive and feed force components exhibited the greatest variability during turning, a comparison was made between their values for the proper machining process—after the tool’s run-in period (point 1 in Figure 5)—and for machining with a worn tool (point 3 in Figure 5). Figure 12 presents this comparison for the passive force component, while Figure 13 shows it for the feed force component.

For cutting inserts A and B, an increase in the values of all four parameters (*L*, *L_max_*, *TT*, and *V_max_*) was observed, with the most significant increases seen for *L_max_* and *V_max_*, which rose by up to 6 to 10 times. However, for cutting insert C, no significant differences in these parameter values were observed. This is most likely because cutting inserts A and B are coated, while insert C is uncoated. As a result of friction during the turning process, the coating layer on inserts A and B is gradually removed, leading to a change in the material interaction between the cutting tool, the workpiece, and the chip. This change alters the dynamics of the cutting process, which is reflected in the recurrence graphs and RQA parameters (*L*, *L_max_*, *TT*, and *V_max_*).

Following the thesis put forward by Radhakrishnan et al. [35], the above studies also confirm that some of the RQA parameters can be the basis for online tool wear state analysis. This suggests that the analysis of recurrence graphs and especially RQA parameters is a useful method for assessing the dynamics of the turning process when machining with coated inserts. However, this method does not appear to be as effective when machining with uncoated inserts. To confirm this conclusion, further experiments should be conducted where the microgeometry of coated and uncoated inserts is kept identical, which is a challenging task due to technological constraints.

One of the potential future applications of the RQA-based methods used in this study is their integration into real-time monitoring systems for tool wear in industrial environments. The ability of RQA parameters to reflect changes in tool wear, as demonstrated in this study, suggests that such methods could be effectively integrated into automated systems. However, several challenges must be addressed before practical implementation is possible. One major challenge is the computational complexity involved in generating recurrence plots and calculating RQA parameters in real time, which requires efficient data processing algorithms. Additionally, the system would need to be robust to variations in cutting conditions and material properties, ensuring accurate tool wear detection without false positives. Lastly, the integration of sensor technologies and real-time data acquisition systems poses technical and cost-related challenges that need to be resolved. Further research is needed to optimize these methods for real-time applications and to validate them under industrial conditions.

## 4. Conclusions

This study analyzes the challenges associated with tool wear during the turning of titanium alloys under MQL conditions, particularly in the context of using different cutting inserts and process analysis methods. Machining of titanium alloys, including Ti-6Al-4V alloy, presents several difficulties due to factors like high strength at elevated temperatures, low thermal conductivity, the tendency for work hardening during cutting, and high chemical reactivity. The issue of tool exploitation and process reliability is crucial in ensuring consistent machining performance, as it directly influences tool longevity and process stability, especially when dealing with hard-to-machine materials. Therefore, the selection of appropriate tools, cutting parameters, and cooling strategies (such as MQL) is critical to the machining efficiency.

Experimentally, turning tests performed under MQL conditions and with different cutting inserts showed significant differences in tool life. The uncoated insert (C) exhibited a much longer tool life compared to coated inserts (A and B), likely due to its sharper geometry.

Recurrence Plot (RP) and Recurrence Quantification Analysis (RQA) methods are effective tools for assessing the dynamics of the cutting process. For coated cutting inserts (A and B), the analysis of these parameters revealed clear changes in process dynamics, particularly related to tool wear and rising cutting forces. In contrast, no similar changes were observed with the uncoated insert (C), suggesting that the RQA method might be more effective for coated tools. Additionally, the study showed that RQA parameters (such as *L_max_*, *V_max_*, and *TT*) combined with recurrence graphs can help identify the critical point of tool wear, particularly for coated tools, although further experiments are needed.

In conclusion, the analysis of recurrence graphs and RQA parameters can be a valuable tool for assessing the dynamics of machining processes, but further investigations are needed, especially for uncoated cutting tools.

## Figures and Tables

**Table 1:** Summary of experimental conditions and turning parameters.

Experimental Conditions		Turning Parameters	
Machine	NEF 600 lathe	Cutting speed *v_c_*, m/min	120
Tool holder	SVJBL2525M16 JET	Depth of cut *a_p_*, mm	0.25
Cutting inserts	VBGT160404 1115 VBGT160404-M3 HX VCMT160404 IC907	Feed *f*, mm/rev	0.1
Workpiece material	Ti-6Al-4V	Fluid flow, mL/h	30
Cutting conditions	MQL	Air pressure, MPa	0.7

## Data Availability

The original contributions presented in this study are included in the article. Further inquiries can be directed to the corresponding author.
